# Effect of pretreatment atmosphere of WO_*x*_/SiO_2_ catalysts on metathesis of ethylene and 2-butene to propylene

**DOI:** 10.1039/c8ra01093e

**Published:** 2018-03-27

**Authors:** Krittidech Gayapan, Sirada Sripinun, Joongjai Panpranot, Piyasan Praserthdam, Suttichai Assabumrungrat

**Affiliations:** Center of Excellence in Catalysis and Catalytic Reaction Engineering, Department of Chemical Engineering, Faculty of Engineering, Chulalongkorn University Bangkok 10330 Thailand suttichai.a@chula.ac.th +66-2218-6877 +66-2218-6868

## Abstract

The effect of a gas pretreatment atmosphere (pure N_2_, pure H_2_ and mixed H_2_/N_2_) on the metathesis reaction between ethylene and 2-butene to propylene over calcined and non-calcined WO_3_/SiO_2_ catalysts was investigated. The non-calcined catalysts exhibited higher activity than the calcined catalysts under different gas pretreatment atmospheres. The non-calcined catalyst with the use of pure H_2_ pretreatment showed the highest catalytic performances. As revealed by various characterization results from N_2_ physisorption, XRD, XPS, TEM, SEM-EDX, UV-Vis, Raman, H_2_-TPR, and NH_3_-TPD techniques, the WO_2.83_ phase occurring from the H_2_ pretreatment of the non-calcined catalyst played an important role on the high activity of the catalyst. In addition, better tungsten dispersion, higher isolated surface tetrahedral tungsten oxide species, and W^5+^ species were obtained on the H_2_-treated non-calcined WO_3_/SiO_2_ catalyst.

## Abstract 2

Effect of H_2_ pretreatment on the non-calcined and air-calcined WO_3_/SiO_2_ catalysts.

## Graphical abstract

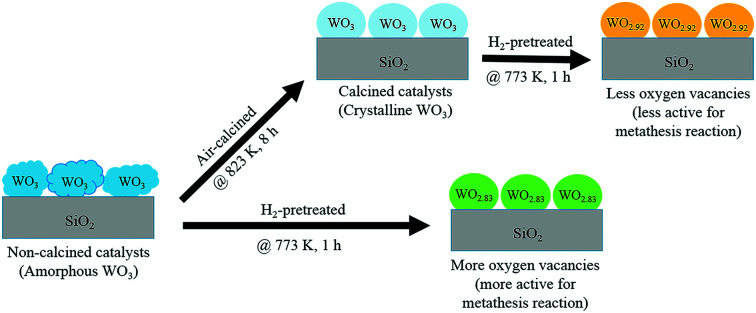

## 1. Introduction

Light olefins have played an important role in the petrochemical industry. In the past, the most important raw material in the petrochemical industry was ethylene and the second one was propylene.^1^ However, nowadays propylene demand has increased since it is used for production of organic chemicals such as polypropylene, acrylonitrile, propylene oxide, alcohol and acrylic acid.^2^ Propylene production can be obtained from several processes such as catalytic and thermal crackers,^3^ methanol to olefins,^4^ propane dehydrogenation^5^ and olefin metathesis.^6^ At present, the metathesis of light olefins has become of particular interest because it could regulate the stocks of light olefins (ethylene, propylene, and butene) upon the market demand at low energy and environmental cost.^7^ Supported tungsten oxide catalysts (especially, WO_3_/SiO_2_) are the most widely used catalysts because of their better resistance to poisoning, lower price, better stability and easy regeneration.^8^ The metathesis of ethylene and 2-butene to propylene on WO_3_/SiO_2_ catalyst has been commercialized as the olefin conversion technology (OCT).^2^ The reaction takes place in a fixed-bed reactor at a temperature > 553 K and pressure of 3.0–3.5 MPa. Since then, many studies have been carried out to develop WO_3_/SiO_2_ catalysts with improved performances. The mechanism of the metathesis reaction of ethylene and 2-butene over WO_3_/SiO_2_ catalysts involves the formation of W–carbene species on the WO_3_ surface, followed by metathesis on these active sites to propylene product.^9^ Many factors affect catalytic activity including the content of tungsten oxide loading,^10^ oxidation state of tungsten species,^11^ conditions of preparation,^12–14^ properties of support,^8,15^ and pretreatment conditions.^16,17^ Experimental studies have reported that the tetrahedral tungsten oxide species are the active sites for metathesis^2,18^ and that WO_3_ crystals are not active in metathesis and catalyst sites should be contained in the amorphous surface.^19^

In order to improve performance of supported WO_*x*_/SiO_2_ catalysts in metathesis reaction, the effect of pretreatment has been investigated. Conventional pretreatment in most studies involves high temperature calcination and inert gas purging.^20,21^ Choung and Weller^22^ showed that some intermediate nonstoichiometric tungsten oxide (WO_3−*x*_) occurring from N_2_ or H_2_ gas pretreatments on WO_3_/SiO_2_ catalysts was the most active species for propylene self-metathesis. Westhoff and Moulijn^23^ found that the slight reduction by H_2_ over WO_3_/SiO_2_ catalysts exhibited activity in propylene self-metathesis better than calcined sample and the sample intermediate between WO_3_ and WO_2.95_ (formed by hydrogen treatment) exhibited the maximum activity. Zaki *et al.*^24^ reported that the formation of WO_3_ catalysts intermediates in the forms of WO_2.96_, WO_2.9_, and WO_2.72_ oxidation states could be adjusted by controlling the H_2_ reduction conditions. Liu *et al.*^17^ studied effect of gas pretreatments including N_2_, mixed H_2_/N_2_, H_2_ and air over the mixed catalysts between MgO and WO_3_/SiO_2_ catalysts on metathesis reaction of 1-butene and ethylene. The results showed that H_2_ content for pretreatment caused the catalyst transformed to the tetragonal WO_3_ and partially reduced W_2.92_, which were active phases for the reaction.

From the previous studies mentioned above, all the researchers used the air-calcined catalysts for further pretreatment or experimental study. To the best of our knowledge, the effect of pretreatment atmosphere on the non-calcined catalysts has not been reported yet. In addition, most studies reported in the literatures performed the reaction tests under atmospheric pressure and high temperature around 723 K,^10–14,25^ while study on high pressure operation was scarce. In this research, the calcined and non-calcined WO_3_/SiO_2_ catalysts for metathesis of ethylene and 2-butene to propylene were studied by using the various gas pretreatments (pure N_2_, pure H_2_ and mixed H_2_/N_2_). The catalytic activity was performed at the temperature of 623 K under low and high pressure conditions at 0.1 and 2.1 MPa. The properties of pretreated catalysts were characterized by the Brunauer–Emmett–Teller (BET) method of N_2_ physisorption, X-ray diffraction (XRD), X-ray photoelectron spectroscopy (XPS), transmission electron microscopy (TEM), scanning electron microscopy (SEM) together with energy dispersive X-ray (EDX) spectroscopy, diffuse reflectance ultraviolet-visible spectra (UV-Vis DRS), Raman microscopy, temperature-programmed reduction of hydrogen (H_2_-TPR) and temperature-programmed desorption of ammonia (NH_3_-TPD) to investigate the relationship of activity, crystallinity, dispersion and interaction of tungsten on the support, and acidity of the catalysts.

## 2. Experimental

### 2.1 Catalyst preparation

The 9 wt% WO_3_/SiO_2_ catalysts were prepared by wetness impregnation using an aqueous solution of ammonium metatungstate hydrate (Aldrich, 99.9%) over silica gel (Davisil Grade 646, 40–60 mesh, supplied by Aldrich). The impregnated sample was dried at room temperature for 2 h and subsequently in an oven at 383 K for 24 h. The catalysts were grouped into two types as the calcined and non-calcined catalysts before pretreatment and reaction testing. For calcined catalysts, the samples were calcined at temperature of 823 K in air for 8 h and then the catalysts were packed in a fixed bed reactor for further pretreatment and reaction. For non-calcined catalysts, the samples after drying were packed in a fixed bed reactor for further pretreatment and reaction.

### 2.2 Pretreatment atmosphere and catalytic evaluation

WO_3_/SiO_2_ catalyst of 3 g was packed at the center of the stainless steel tubular fixed-bed reactor (internal diameter = 19.05 mm). The catalyst was heated to 773 K by a furnace under N_2_ gas at a flow rate of 30 ml min^−1^ and held at 773 K for 1 h. After that, pretreatment gas (pure N_2_, pure H_2_ or mixed 1 : 1 H_2_/N_2_) at a total flow rate of 30 cm^3^ min^−1^ was fed to the reactor for 1 h. The reactor was then cooled down to operating temperature of 623 K under N_2_ gas at the same flow rate. The reaction was started by introducing a feed containing 4% 2-butene (2% *cis*-2-butene and 2% *trans*-2-butene) and 8% ethylene balanced in nitrogen gas. The reaction condition was kept at 623 K with a WHSV of 0.52 h^−1^. The operating pressure was varied at 0.1 and 2.1 MPa. The catalysts were denoted as W-cal-fresh and W-noncal-fresh for calcined and non-calcined fresh catalysts, but catalysts with different gas pretreatments were denoted as W-cal-xxx and W-noncal-xxx represented the calcined and non-calcined catalyst where “xxx” represents the gas pretreatment including N_2_, H_2_ and H_2_/N_2_ for gas pretreatment with pure N_2_, pure H_2_ and mixed H_2_/N_2_, respectively.

**Scheme 1:**
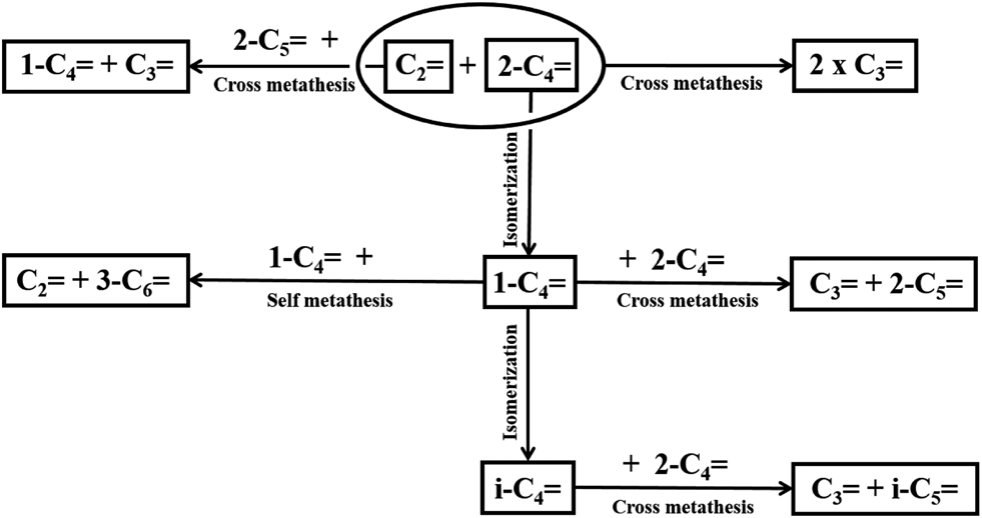
Reaction pathways of ethylene and 2-butene.

The products from the reaction tests were analyzed by using an online gas chromatography (Agilent 7820A), which was equipped with a flame ionization detector (FID). The reaction pathways were illustrated in Scheme 1 in details.^26,27^ The main reaction was the metathesis of ethylene and 2-butene to produce propylene as the conventional reaction for propylene production in industrial plants and the side reactions included isomerization, cross-metathesis and self-metathesis reaction as shown in this scheme. The 2-butene conversion, product selectivity and yields were determined by the following equations:2-Butene conversion = ((amount of *trans*- and *cis*-2-butene in feed − amount of *trans*- and *cis*-2-butene in products)/amount of *trans*- and *cis*-2-butene in feed) × 100Product selectivity = (amount of any product/amount of total product) × 100Product yield = (2-butene conversion × product selectivity)/100

### 2.3 Catalyst characterization

XRD patterns of the catalysts were determined by using a D8 Advance of Bruker AXS using Ni-filter Cu K_α_ radiation in the 2*θ* range of 20° to 80°. For phase composition identification purposes, the diffraction patterns were matched with standard diffraction data files. The BET surface areas, pore volumes and pore sizes were obtained using Micromeritics Chemisorbs 2750 with nitrogen adsorption studies. The surface structures of tungsten oxide species were examined by Raman microscopy under ambient conditions using a Senterra Dispersive Raman Microscopy (Bruker Optics) equipped with the laser wavelength at 532 nm and a TE-cooled CCD detector. The UV-Vis DRS was used to investigate surface structure of tungsten oxide species. The catalyst samples were recorded on Lambda 650 spectrophotometer in the range between 200 and 800 nm. The XPS was carried out using an AMICUS photoelectron spectrometer equipped with an Mg K_α_ X-ray as a primary excitation and KRATOS VISION2 software. XPS elemental spectra were acquired with 0.1 eV energy step at a pass energy of 75 eV.

The TEM images were used to investigate the particle morphology and lattice spacing of the samples, which was conducted on a JEOL JEM-2010 microscope equipped with a LaB_6_ electron gun in the voltage range of 200 kV. TEM samples were prepared by dispersing in ethanol by sonication and a few drops of suspension onto a carbon-coated copper grid followed by solvent evaporation in air at room temperature. The tungsten distribution over the silica support were investigated by SEM (Hitachi S3400N) equipped with EDX (EDAX Apollo-X).

H_2_-TPR was performed to investigate the reducibility of catalysts by a Micromeritics Chemisorb 2750 automated system. The catalyst was loaded into a quartz U-tube reactor. Prior to H_2_-TPR experiment, the sample was pretreated under N_2_ flow at 773 K for 1 h and then cooled to 313 K. Subsequently, the catalyst was reduced by using 10% H_2_/Ar with a flow rate of 25 ml min^−1^ at a heating rate of 5 K min^−1^ from 313 to 1173 K. The amount of hydrogen uptake was determined by measuring the areas of the reduction profiles on the thermal conductivity detector (TCD).

NH_3_-TPD was used to determine the acidity of catalysts by a Micromeritics Chemisorb 2750 automated system. The catalyst packed in a quartz U-tube reactor was pretreated with a He flow rate of 25 ml min^−1^ at 773 K for 1 h and then cooled down to 313 K. After that, it was exposed to a 15% NH_3_/He mixed gas with a flow rate of 25 ml min^−1^ for 30 min. Subsequently, the sample was purged with a He gas stream (25 ml min^−1^) for 1 h and the temperature was increased linearly with a rate of 5 K min^−1^ to 773 K. The desorbed ammonia was detected by using the TCD.

## 3. Results and discussion

### 3.1 Catalytic performances of the calcined and non-calcined catalysts

The metathesis performance was measured for the calcined and non-calcined catalysts in metathesis of 2-butene (*trans*- and *cis*-) and ethylene to produce propylene with different gas pretreatments. The reaction conditions were operated at 623 K, 0.52 h^−1^ of WHSV at two pressures (0.1 and 2.1 MPa) with the reactant of 2% *cis*-2-butene, 2% *trans*-2-butene and 8% ethylene in N_2_ balanced, and the reaction tests were terminated after 12 h. Table 1 shows the activity and product yields of the calcined and non-calcined catalysts with different gas pretreatment atmospheres for operation of 1 h. The 2-butene conversion at 0.1 MPa for the calcined catalysts with different gas pretreatments was around 12–23%, while those of the non-calcined catalysts were around 22–24%. For operation at 2.1 MPa, the 2-butene conversion for the calcined and non-calcined catalysts were around 24–26% and 48–69%, respectively. These results showed that the non-calcined catalysts exhibited the 2-butene conversion higher than the calcined catalysts. Considering the effect of gas pretreatment at pressure of 0.1 MPa, the increase of H_2_ content in the gas pretreatment was likely to increase 2-butene conversion in both calcined and non-calcined catalysts. At pressure of 2.1 MPa, the difference in 2-butene conversion with increasing H_2_ content between the calcined and non-calcined catalysts was more pronounced. The pure H_2_ pretreatment of the non-calcined catalyst exhibited the highest 2-butene conversion. Considering the product yields, when comparing the propylene and 1-butene yields, the non-calcined catalysts also exhibited the propylene yield higher than the calcined catalysts, while the 1-butene yield for the non-calcined catalysts was lower than the calcined catalysts at both pressures. In addition, when considering isobutene and C_5_^+^= yields, the non-calcined catalysts were higher than the calcined catalysts. Comparing among different gas pretreatments, the use of pure H_2_ for pretreatment increased the propylene yield for both 0.1 and 2.1 MPa pressures, while the yields of side reaction products such as 1-butene, isobutene, and C_5_^+^= were slightly decreased, except for the pure N_2_ pretreatment of the calcined catalyst that the side product yields were lower than the others.

**Table 1:** 2-Butene conversion and product yields of the calcined and non-calcined WO_3_/SiO_2_ catalysts under different gas pretreatment atmospheres at pressure of 0.1 and 2.1 MPa^a^

Catalysts	2-Butene conversion (%)		Yield (%) at 0.1 MPa				Yield (%) at 2.1 MPa			
	0.1 MPa	2.1 MPa	Propylene	1-Butene	Isobutene	C_5_^+^=	Propylene	1-Butene	Isobutene	C_5_^+^=
W-noncal-N_2_	22.3	48.4	0.71	21.1	0.32	0.18	35.3	7.41	1.55	4.05
W-noncal-H_2_/N_2_	23.4	66.6	1.10	20.8	1.25	0.23	56.7	5.05	1.33	3.60
W-noncal-H_2_	23.7	68.8	1.31	20.8	1.38	0.18	59.3	5.05	1.02	3.46
W-cal-N_2_	12.4	24.6	0.32	11.8	0.09	0.18	8.10	15.4	0.42	0.70
W-cal-H_2_/N_2_	22.7	45.3	0.31	21.4	0.79	0.15	33.2	8.14	1.18	2.71
W-cal-H_2_	22.8	46.2	0.40	21.3	0.95	0.18	34.8	7.82	1.02	2.58

a Reaction conditions: *T* = 623 K; WHSV = 0.52 h^−1^; time on stream = 1 h.

**Fig. 1:**
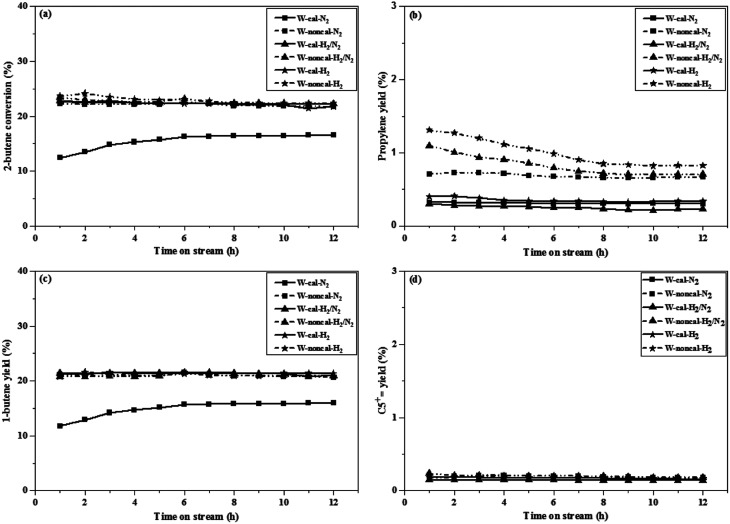
2-Butene conversion and product yields of the calcined (continuous lines) and non-calcined (dotted lines) WO_3_/SiO_2_ catalysts under different pressures and gas pretreatments at pressure of 0.1 MPa: (a) 2-butene conversion, (b) propylene yield, (c) 1-butene yield and (d) C_5_^+^= yield (reaction condition: *T* = 623 K; WHSV = 0.52 h^−1^).

**Fig. 2:**
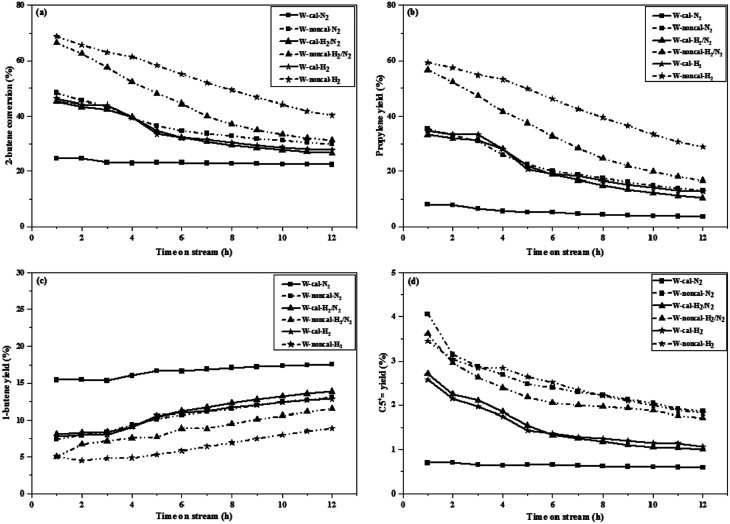
2-Butene conversion and product yields of the calcined (continuous lines) and non-calcined (dotted lines) WO_3_/SiO_2_ catalysts under different pressures and gas pretreatments at pressure of 2.1 MPa: (a) 2-butene conversion, (b) propylene yield, (c) 1-butene yield and (d) C_5_^+^= yield (reaction condition: *T* = 623 K; WHSV = 0.52 h^−1^).

The 2-butene conversion and product yields at operation of 0.1 and 2.1 MPa as a function of time on stream for 12 h were presented in Fig. 1 and 2, respectively. For operation at 0.1 MPa as shown in Fig. 1, the 2-butene conversion was unchanged among the different gas pretreatments for both calcined and non-calcined catalysts and rather constant through 12 h time-on-stream, except the case of N_2_ pretreatment of the calcined catalyst, it showed the lower 2-butene conversion than the other pretreatments. However, the 2-butene conversion of the non-calcined catalysts was better than the calcined samples. The propylene yields (see Fig. 1b) of the catalysts followed the order: W-noncal-H_2_ > W-noncal-H_2_/N_2_ > W-noncal-N_2_ > W-cal-H_2_ > W-cal-H_2_/N_2_ > W-cal-N_2_, while the 1-butene yield (see Fig. 1c) indicated the opposite trend to that of the propylene yield. In addition, the C_5_^+^ yield (see Fig. 1d) was very low and insignificantly different for all the catalysts. Nevertheless, the propylene yield of the non-calcined catalysts decreased initially and became constant after 8 h, whereas 1-butene yield of the non-calcined catalyst was slightly constant. When operating at pressure of 2.1 MPa as shown in Fig. 2, the differences in 2-butene conversion and product yields of the calcined and non-calcined catalysts were more pronounced. The non-calcined catalysts exhibited the activity of 2-butene conversion and propylene yield higher than the calcined samples, while the 1-butene yield of the non-calcined catalysts was lower than the calcined catalyst and the C_5_^+^= yield of the non-calcined catalysts was higher than the calcined catalysts. Considering the effect of gas pretreatment, it was found that the results of 2.1 MPa were the same trend as the operation at 0.1 MPa. The increasing H_2_ content for pretreatment exhibited the high 2-butene conversion and propylene yield and caused the lower 1-butene yield. Pure H_2_ pretreatment of the non-calcined catalysts exhibited the best catalytic activity and performances for the metathesis of ethylene and 2-butene.

### 3.2 Characterization of the calcined and non-calcined catalysts

The study of pretreatment atmospheres of the WO_3_/SiO_2_ catalysts on metathesis reaction of ethylene and 2-butene showed that the non-calcined catalysts exhibited the activity and performance higher than the calcined catalysts and the pure H_2_ pretreatment of the non-calcined catalyst exhibited the highest activity. Different gas pretreatments could lead to the changes of the dispersion of tungsten species, the interaction of the tungsten and support, the structure of surface tungsten compounds, and the partial oxidation state of the catalysts, which played an important role in activity of metathesis reaction. The calcined and non-calcined WO_3_/SiO_2_ catalysts pretreated under different gas atmospheres were characterized by BET, XRD, XPS, TEM, SEM-EDX, UV-Vis, Raman, H_2_-TPR and NH_3_-TPD techniques.

#### 3.2.1 N_2_ physisorption

The BET surface areas, pore volumes, and average pore sizes of fresh catalysts obtained from different gas pretreatments were analyzed and the results are shown in Table 2. It was found that the calcined and non-calcined catalysts showed no significant differences in the specific surface area, pore volume, and average pore size. In other words, the gas pretreatment did not affect the physical properties of the calcined and non-calcined catalysts.

**Table 2:** BET surface areas, pore volumes and pore sizes of the calcined and non-calcined WO_3_/SiO_2_ catalysts for different gas pretreatment atmospheres

Catalysts	BET surface area (m^2^ g^−1^)	Pore volume (cm^3^ g^−1^)	Pore size (nm)
W-noncal-fresh	288	1.01	9.51
W-noncal-N_2_	296	1.02	9.50
W-noncal-H_2_/N_2_	303	1.03	9.39
W-noncal-H_2_	298	1.03	9.45
W-cal-fresh	285	0.97	9.41
W-cal-N_2_	286	1.02	9.15
W-cal-H_2_/N_2_	288	1.00	9.39
W-cal-H_2_	287	1.04	9.21

#### 3.2.2 X-ray diffraction (XRD)

**Fig. 3:**
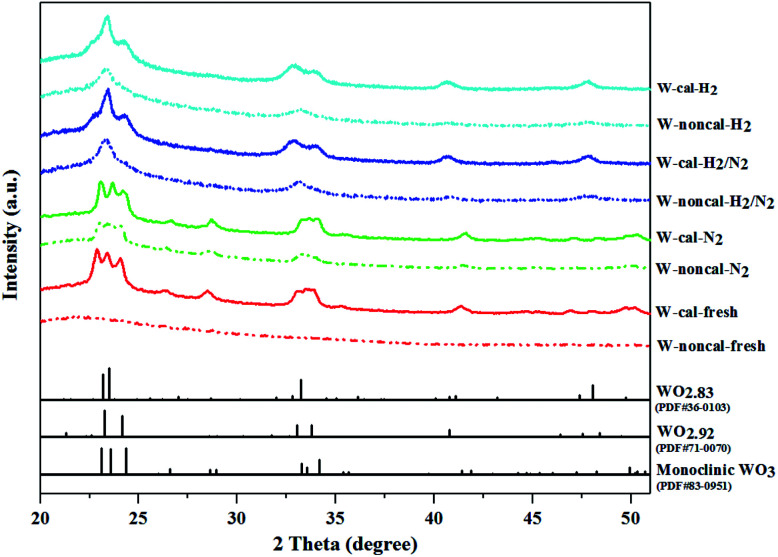
XRD patterns of the calcined (colored continuous lines) and non-calcined (dotted lines) WO_3_/SiO_2_ catalysts with different gas pretreatments and standard data reference from JCPDS (black continuous lines).

The XRD diffraction patterns of catalysts with different gas pretreatments are shown in Fig. 3. The as-prepared non-calcined WO_3_/SiO_2_ catalyst (W-noncal-fresh) showed no obvious peaks corresponding to crystalline phase of WO_3_, indicating that the W phase was well dispersed on the supports forming an amorphous phase.^10^ The fresh catalyst after calcination (W-cal-fresh) at 823 K for 8 h exhibited XRD patterns assigned to monoclinic WO_3_. When the catalysts (calcined and non-calcined) were pretreated with different gases including pure N_2_ (W-xxx-N_2_), mixed H_2_/N_2_ (W-xxx-H_2_/N_2_), and pure H_2_ (W-xxx-H_2_), the diffraction peaks based on standard data reference in JCPDS showed different patterns. Pure N_2_ pretreatment for the calcined and non-calcined catalysts exhibited the same XRD patterns as monoclinic WO_3_, but the patterns of the non-calcined catalysts exhibited a lower crystallinity than those of the calcined catalysts, indicating the better dispersed tungsten species on the surface.^25^ As shown in the activity results (Fig. 1 and 2), the N_2_ pretreatment of the non-calcined catalyst exhibited higher activity than the calcined catalyst, indicating that less WO_3_ crystallinity was active in metathesis.^19^ For the mixed H_2_/N_2_ and pure H_2_, the non-calcined catalysts exhibited the patterns assigned to WO_2.83_ phase, while the calcined catalysts exhibited WO_2.92_ phase, occurring from the partial reduction of WO_3_.^17^ The published literatures reported that the partial reduction or intermediate of tungsten oxide on support affected the metathesis activity. Westhoff *et al.*^23^ proposed that the intermediate between WO_3_ and WO_2.95_ was the active phase for metathesis reaction and Choung *et al.*^22^ showed some intermediate WO_3−*x*_ to be the most active for propylene metathesis, while Liu *et al.*^17^ indicated that the tetragonal WO_3_ and partially reduced WO_2.92_ were the active phases for metathesis. In our results (Fig. 1 and 2), we found that H_2_ pretreatment of the non-calcined catalyst exhibited higher activity than the calcined catalysts. Therefore, it could be proposed that WO_2.83_ crystalline phase of the catalysts played an important role on the active phase in the metathesis reaction of ethylene and 2-butene. When comparing between using mixed H_2_/N_2_ and pure H_2_ pretreatment, the pure H_2_ pretreatment exhibited the lower crystallinity than the mixed H_2_/N_2_ pretreatment. Therefore, the pure H_2_ pretreatment offered the better dispersion of W species on the support and that of the non-calcined catalyst showed the best dispersion.

#### 3.2.3 X-ray photoelectron spectroscopy (XPS)

**Fig. 4:**
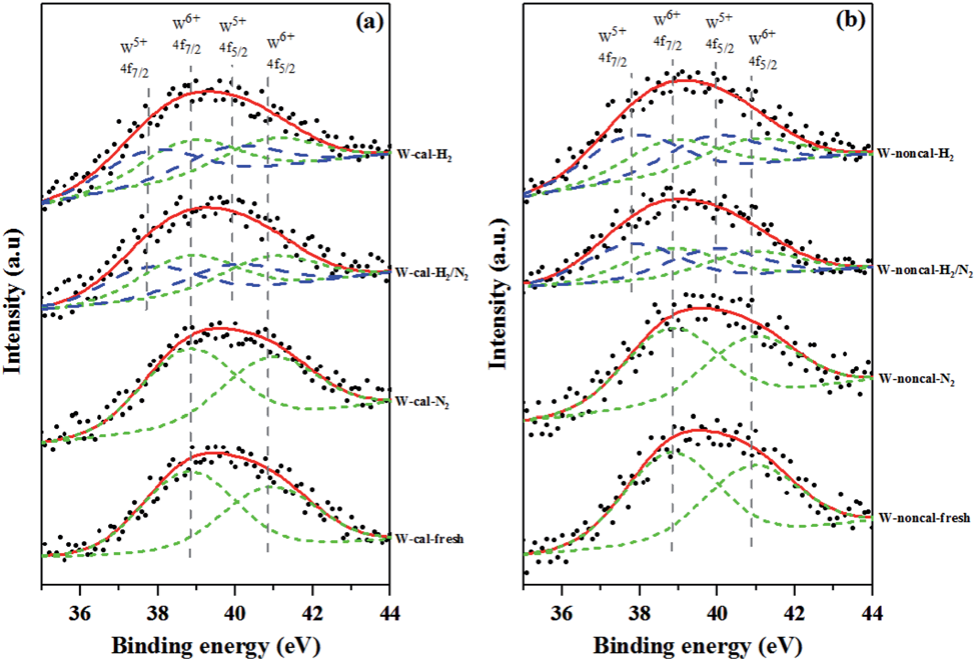
XPS spectra of the calcined (a) and non-calcined (b) WO_3_/SiO_2_ catalysts with different gas pretreatments.

XPS measurements were used to determine the surface chemical state of tungsten on the surface of the calcined and non-calcined WO_3_/SiO_2_ catalysts obtained with various gas pretreatments and the results are shown in Fig. 4. The deconvoluted peaks were based on the full width at half-maximum (FWHM) of 1.4 eV, the binding energy difference between W 4f_5/2_ and W 4f_7/2_ of 2.1 eV, the peak intensity ratio of W 4f_5/2_ and W 4f_7/2_ (*I*(f_7/2_) : *I*(f_5/2_)) of 4 : 3, and the binding energy difference between W^5+^ 4f_7/2_ and W^6+^ 4f_7/2_ peak about 0.9–1.1 eV.^28,29^ The binding energies of 38.8 and 40.9 eV were assigned to W^6+^ 4f_7/2_ and W^6+^ 4f_5/2_, while the ones of 37.8 and 39.9 eV were assigned to W^5+^ 4f_7/2_ and W^5+^ 4f_5/2_. The fresh and pure N_2_ pretreatment of the calcined and non-calcined catalysts exhibited only W^6+^ phase, indicating WO_3_ on the silica support. While the mixed H_2_/N_2_ and pure H_2_ pretreatment exhibited W^5+^ and W^6+^ phases, indicating the WO_3−*x*_ on the silica support.^30^ As shown in the figure, the XPS analysis for the mixed H_2_/N_2_ and pure H_2_ pretreatment confirmed that the W^6+^ was converted to W^5+^ and that the amount of the W^5+^ oxidation state increased with increasing hydrogen in the gas pretreatment. Nevertheless, it was found that the mixed H_2_/N_2_ and pure H_2_ pretreatment on the non-calcined catalysts exhibited the W^5+^ peak higher than the calcined samples (see Table 2). Such results indicated the higher degree of W^6+^ conversion to W^5+^ state, which was consistent with the XRD results that showed WO_2.83_ phase for the non-calcined catalyst and WO_2.92_ phase for the calcined catalyst.

The surface compositions of the calcined and non-calcined WO_3_/SiO_2_ catalysts were determined by XPS and the results are shown in Table 3. All the catalysts showed insignificant changes of oxygen and carbon content on the catalyst surface whereas silica and tungsten compounds were different on the calcined and the non-calcined catalysts. Considering the ratios of W/(Si + W), which indicated the surface dispersion and concentration of tungsten on silica support,^31^ the non-calcined catalysts showed higher ratios of W/(Si + W) than those of the calcined samples. It is suggested that the W surface concentration on the non-calcined catalysts was higher than the calcined catalysts, which was consistent to the XRD results. There was little effect of the gas pretreatment atmosphere on the dispersion of W species for both inside the pores and on the outer surface of the catalysts.

**Table 3:** Surface characterization by X-ray photoelectron spectroscopy (XPS) on the calcined and non-calcined WO_3_/SiO_2_ catalysts with different gas pretreatments

Catalysts	Elements (at%)				W/(Si + W)	W^5+^ (%)	W^6+^ (%)
	O	C	Si	W			
W-noncal-fresh	78.1	4.66	10.6	6.57	0.38	0.00	100.0
W-noncal-N_2_	78.5	4.57	11.1	5.83	0.35	0.00	100.0
W-noncal-H_2_/N_2_	78.5	3.48	11.5	6.53	0.36	54.7	45.3
W-noncal-H_2_	79.0	3.91	11.2	5.89	0.35	56.6	43.4
W-cal-fresh	79.1	3.62	11.7	5.56	0.32	0.00	100.0
W-cal-N_2_	78.6	3.24	12.1	6.01	0.33	0.00	100.0
W-cal-H_2_/N_2_	79.2	2.46	12.1	6.23	0.34	45.0	55.0
W-cal-H_2_	78.5	3.55	12.0	5.89	0.33	47.2	52.8

#### 3.2.4 Transmission electron microscopy (TEM)

**Fig. 5:**
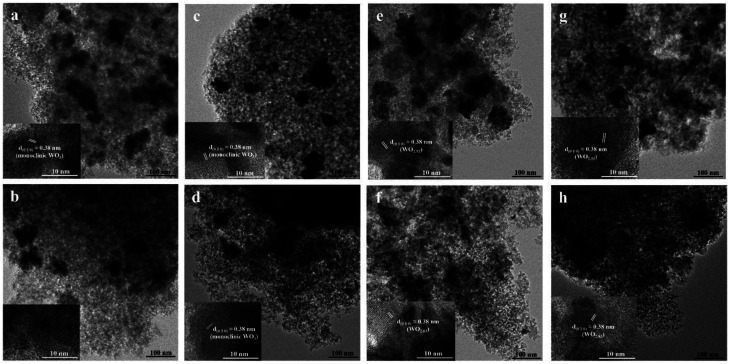
TEM images and high-resolution TEM image (inset) of the calcined (top) and non-calcined (bottom) WO_3_/SiO_2_ catalysts with different gas pretreatments; (a) W-cal-fresh, (b) W-noncal-fresh, (c) W-cal-N_2_, (d) W-noncal-N_2_, (e) W-cal-H_2_/N_2_, (f) W-noncal-H_2_/N_2_, (g) W-cal-H_2_ and (h) W-noncal-H_2_.

The particle morphology and lattice spacing of the samples were investigated by TEM and high resolution TEM images as shown in Fig. 5. It was found that the calcined catalysts showed more tungsten agglomerates dispersed on the surface of silica than the non-calcined catalysts, indicating poor tungsten dispersion of the calcined catalysts. Such results were consistent to the XRD and XPS results. Under different gas pretreatment atmospheres, pure H_2_ pretreatment on the non-calcined catalyst showed the best dispersion of tungsten on the support. From the high resolution TEM images (inset figure), only one crystal lattice plane was observed. The lattice spacing of all the pretreated catalysts was approximately around 0.38 nm, except the fresh non-calcined catalyst, in which the lattice spacing was not found due to its amorphous structure. From the XRD standard data reference in JCPDS, the lattice spacing of the (020) planes of monoclinic WO_3_, (010) planes of WO_2.92_ and (010) planes of WO_2.83_ were about 0.377, 0.382 and 0.379 nm, respectively. Therefore, the lattice spacing obtained from the high resolution TEM images was consistent with the XRD standard data.

#### 3.2.5 Scanning electron microscopy with energy dispersive X-ray spectroscopy (SEM-EDX)

**Fig. 6:**
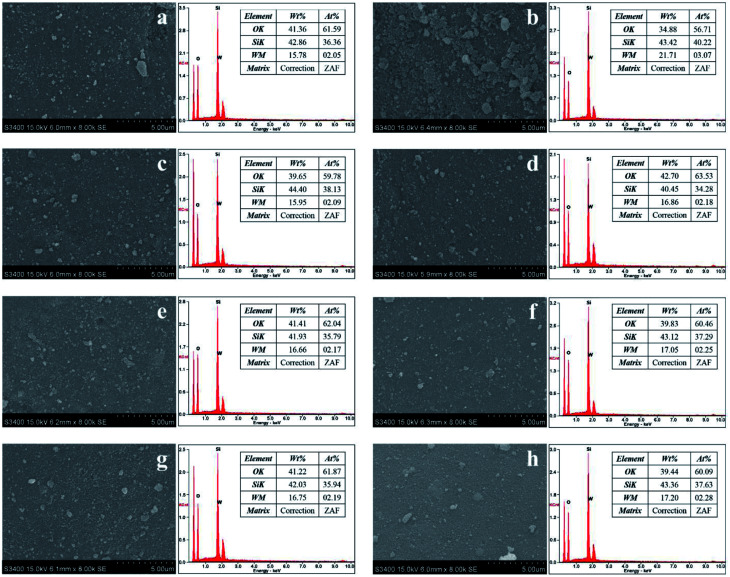
SEM and SEM-EDX images of the calcined (left) and non-calcined (right) WO_3_/SiO_2_ catalysts with different gas pretreatments; (a) W-cal-fresh, (b) W-noncal-fresh, (c) W-cal-N_2_, (d) W-noncal-N_2_, (e) W-cal-H_2_/N_2_, (f) W-noncal-H_2_/N_2_, (g) W-cal-H_2_ and (h) W-noncal-H_2_.

The SEM and SEM-EDX were used to investigate the tungsten distribution over the surface of silica (Fig. 6). The SEM results revealed that the topology of catalysts was non-uniform with irregular shape. The non-calcined catalysts with gas pretreatment showed fewer agglomerates compared to the calcined catalysts. In addition, from the measurement of tungsten on the silica support by SEM-EDX, the non-calcined catalysts showed higher amount of tungsten on the support. Therefore, tungsten dispersion of the non-calcined catalysts was higher than the calcined catalysts, consistent with the XRD, XPS, and TEM results. Under the different gas pretreatment atmospheres, pure H_2_ pretreatment of the non-calcined catalyst offered the best tungsten dispersion on the support.

#### 3.2.6 Diffuse reflectance ultraviolet-visible spectra (UV-Vis DRS)

**Fig. 7:**
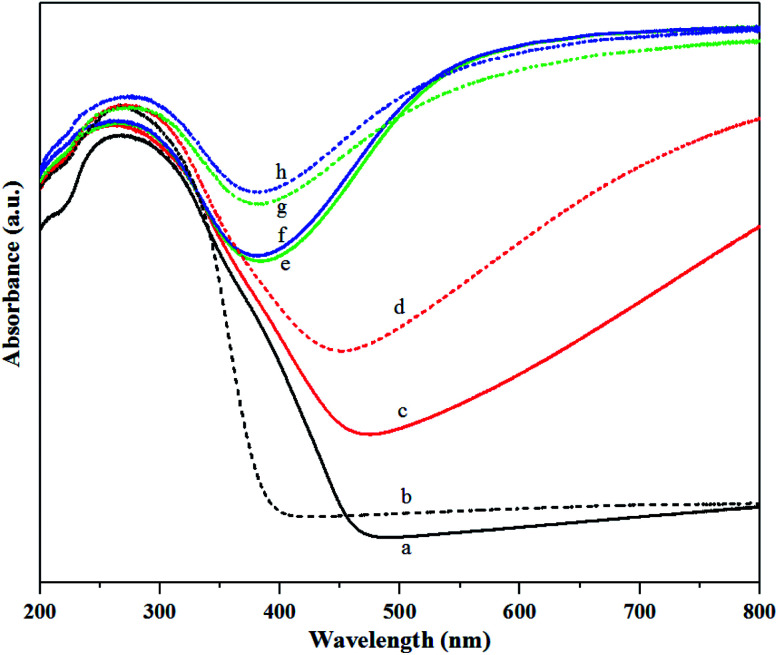
UV-Vis DRS patterns of the calcined (continuous lines) and non-calcined (dotted lines) WO_3_/SiO_2_ catalysts with different gas pretreatments; (a) W-cal-fresh, (b) W-noncal-fresh, (c) W-cal-N_2_, (d) W-noncal-N_2_, (e) W-cal-H_2_/N_2_, (f) W-noncal-H_2_/N_2_, (g) W-cal-H_2_ and (h) W-noncal-H_2_.

Structure of tungsten species produced by different gas pretreatments on the calcined and non-calcined catalysts were analyzed by UV-Vis DRS as shown in Fig. 7. Two absorption bands at 230 and 270 nm were observed in the spectra for all the catalysts, whereas the adsorption band at 400 nm was observed obviously for the fresh calcined catalyst and N_2_ pretreatment of both calcined and non-calcined catalysts. Nevertheless, the adsorption bands between 400 and 800 nm were observed for pure N_2_, pure H_2_ and mixed 1 : 1 H_2_/N_2_ pretreatment for both calcined and non-calcined catalysts. According to the literature,^10,32^ the absorption bands at 230 nm could be assigned to isolated tetrahedral [WO_4_]^2−^ species, while the absorption bands at 270 nm corresponded to octahedral polytungstate species. The band at 400 nm was assigned to WO_3_ crystallites,^10,33^ whereas the adsorption bands between 400 and 800 nm could be assigned to W^4+^ and W^5+^ species.^32^ The adsorption bands at 230 and 270 nm were attributed to W^6+^ species.^10,32^ As shown in Fig. 7, the intensity of adsorption band at 230 nm for all the samples was not much different, except the fresh calcined catalyst in which the band intensity was lower than the others. However, the pure H_2_ pretreatment of the non-calcined catalyst exhibited the highest intensity. For the adsorption band at 270 nm, the non-calcined catalysts exhibited the intensities higher than the calcined catalysts and the pure H_2_ pretreatment of the non-calcined catalyst showed the highest band intensity. Intensity of the band at 400 nm was obviously observed for the fresh calcined catalyst and N_2_ pretreatment of the calcined and non-calcined catalysts. The calcined samples showed more obvious peaks than the non-calcined catalysts, indicating a poor dispersion on silica,^10^ which was consistent with the XRD and XPS results. When pretreating with H_2_ (mixed H_2_/N_2_ and pure H_2_), the adsorption band at 400 nm was converted to adsorption bands between 400 and 800 nm. In other words, the crystalline WO_3_ was reduced by gas pretreatment, which was consistent with the XRD and XPS results. Comparing the cases of mixed H_2_/N_2_ and pure H_2_ pretreatment for the calcined and non-calcined samples showed that the bands between 400 and 800 nm of the non-calcined catalysts were broader than the calcined catalysts, which were ascribed to ordered mesoporous structure and enlarged oxygen vacancies due to higher W^5+^ in sub-stoichiometric WO_3−*x*_.^30^

#### 3.2.7 Raman spectroscopy

**Fig. 8:**
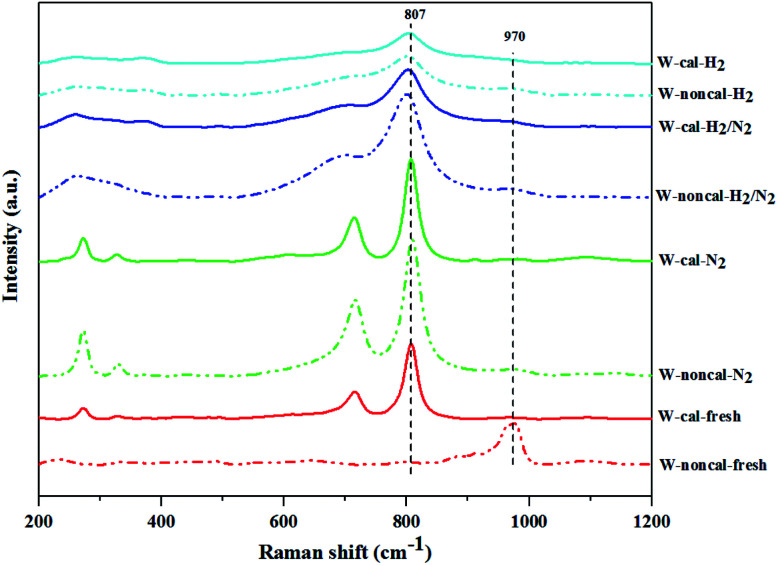
Raman spectra of the calcined (continuous lines) and non-calcined (dotted lines) WO_3_/SiO_2_ catalysts with different gas pretreatments.

The Raman spectroscopy results showing the structure of tungsten species present on the supported catalysts are shown in Fig. 8. The bands at 263–275, 707–720 and 805–808 cm^−1^ were assigned to the deformation mode of W–O–W, bending mode of W–O, and symmetric stretching mode of W–O, respectively.^15,31^ The Raman band at 325–329 cm^−1^ assigned to bending mode of O–W–O of surface WO_*x*_.^8,10^ The broad band at 970 cm^−1^ was assigned to the O=W=O band of isolated surface tetrahedral tungsten oxide species.^31^ Fresh catalyst without calcination (W-noncal-fresh) showed the clear Raman band at 970 cm^−1^ and a small broad peak at 807 cm^−1^, indicating the signature bands of distorted WO_6_ units.^34^ While fresh catalyst with calcination (W-cal-fresh) and N_2_ pretreatment on both calcined and non-calcined catalysts showed the four strongest peaks (263–275, 325–329, 707–720 and 805–808 cm^−1^) of crystalline WO_3_ ^8^ and a broad small peaks at 970 cm^−1^. For the H_2_ pretreatment, the Raman bands at 710 and 807 cm^−1^ became broader as the H_2_ content increased. It is suggested that there was a gradual degradation of the crystallinity upon hydrogen treatment due to an increasing amount of oxygen vacancies in the form of WO_3−*x*_ phase.^30^ Comparing the cases of H_2_ pretreatment for the calcined and non-calcined catalysts, the Raman band at 970 cm^−1^ of non-calcined catalysts showed more obvious peaks than those of the calcined samples, indicating more isolated surface tetrahedral tungsten oxide species as also confirmed by the UV-Vis results. The ratios of relative Raman intensities of the peak at 970 to 807 cm^−1^ (*I*_970_/*I*_807_) are shown in Table 4 as an indicative of the relative amount of isolated surface tetrahedral tungsten oxide species (active site) to WO_3_ crystal (non-active site).^35,36^ The pure H_2_ pretreatment of the non-calcined catalyst exhibited the highest *I*_970_/*I*_807_ ratio among the catalysts studied. Based on the characterization results described above, we can conclude that the H_2_ pretreatment (mixed H_2_/N_2_ and pure H_2_) of the non-calcined catalysts exhibited higher oxygen vacancies to WO_3−*x*_ than the calcined ones and the pure H_2_ pretreatment of the non-calcined catalyst showed the highest amount of isolated surface tetrahedral tungsten oxide.

**Table 4:** Integral of Raman band area at intensity of 807 and 970 cm^−1^ and its relative intensities (*I*_970_/*I*_807_) on the calcined and non-calcined WO_3_/SiO_2_ catalysts with different gas pretreatments

Catalysts	Peak area of intensity (×10^4^)		*I*_970_/*I*_807_ ratio
	807 cm^−1^	970 cm^−1^	
W-noncal-fresh	1.73	25.5	14.8
W-noncal-N_2_	69.2	4.27	0.06
W-noncal-H_2_/N_2_	84.1	5.69	0.07
W-noncal-H_2_	31.5	3.60	0.11
W-cal-fresh	34.6	0.89	0.03
W-cal-N_2_	48.2	1.44	0.03
W-cal-H_2_/N_2_	49.0	2.40	0.05
W-cal-H_2_	18.2	1.19	0.07

#### 3.2.8 Temperature-programmed desorption of ammonia (NH_3_-TPD)

**Fig. 9:**
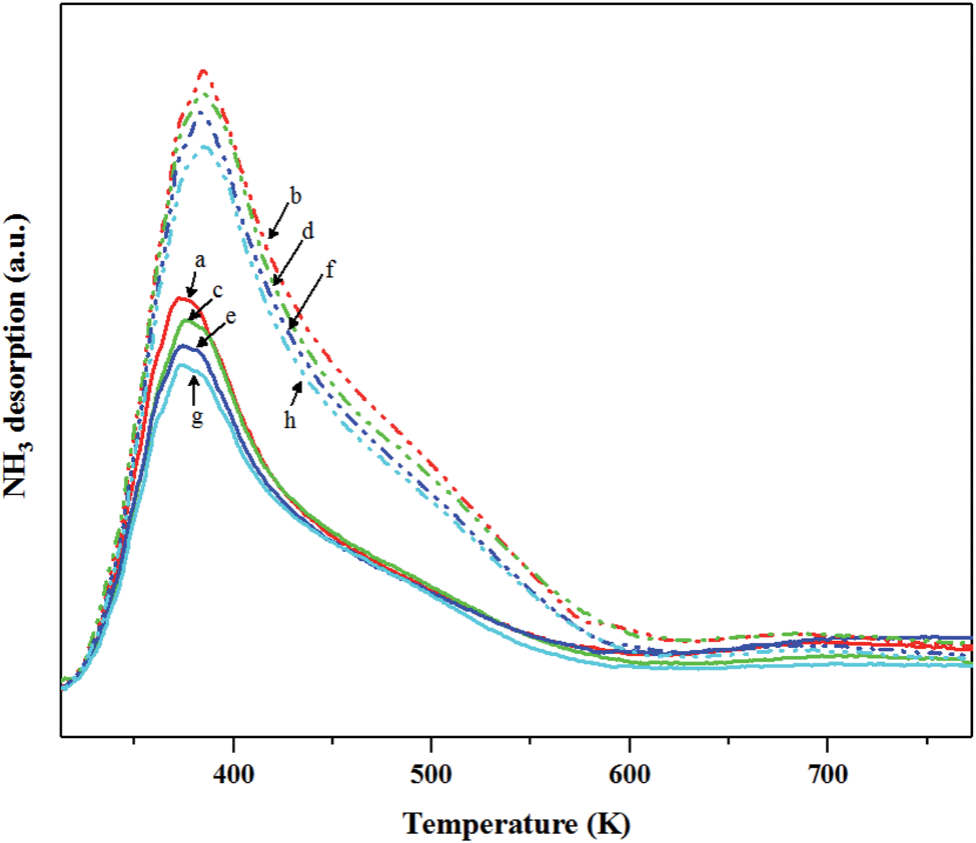
NH_3_-TPD profiles of the calcined (continuous lines) and non-calcined (dotted lines) WO_3_/SiO_2_ catalysts with different gas pretreatments; (a) W-cal-fresh, (b) W-noncal-fresh, (c) W-cal-N_2_, (d) W-noncal-N_2_, (e) W-cal-H_2_/N_2_, (f) W-noncal-H_2_/N_2_, (g) W-cal-H_2_ and (h) W-noncal-H_2_.

**Fig. 10:**
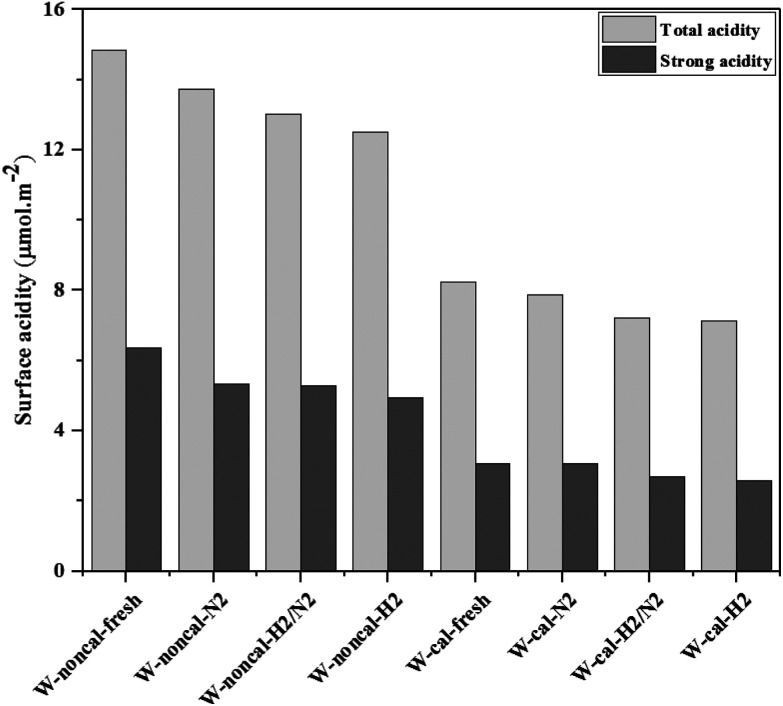
The total and strong acidity of the calcined and non-calcined WO_3_/SiO_2_ catalysts with different gas pretreatments.

The NH_3_-TPD was performed to characterize the surface acidity of the catalysts. In Fig. 9, the ammonia desorption peaks of the non-calcined WO_3_/SiO_2_ catalysts occurred at higher temperature than the calcined catalysts, indicating the higher acid strength^31^ as stronger acidity. The amounts of NH_3_ desorption decreased with changing gas pretreatment as follows; pure N_2_ > mixed H_2_/N_2_ > pure H_2_. In addition, the gas pretreatment caused the ammonia desorption peak to slightly shift to lower temperature, indicating the lower acid strength. This demonstrated that using the gas pretreatment could result in a lower amount of total acid and acid strength. The surface acidity as shown in Fig. 10 also confirmed clearly that gas pretreatment affected to the total acid amount and strength. It appeared that the N_2_ pretreatment exhibited both total acid amount and strong acid strength higher than the mixed H_2_/N_2_ and pure H_2_ pretreatment for both calcined and non-calcined catalysts. In other words, H_2_ content of gas pretreatment caused only a slight change of acid amount and strength.

#### 3.2.9 Temperature-programmed reduction of hydrogen (H_2_-TPR)

**Fig. 11:**
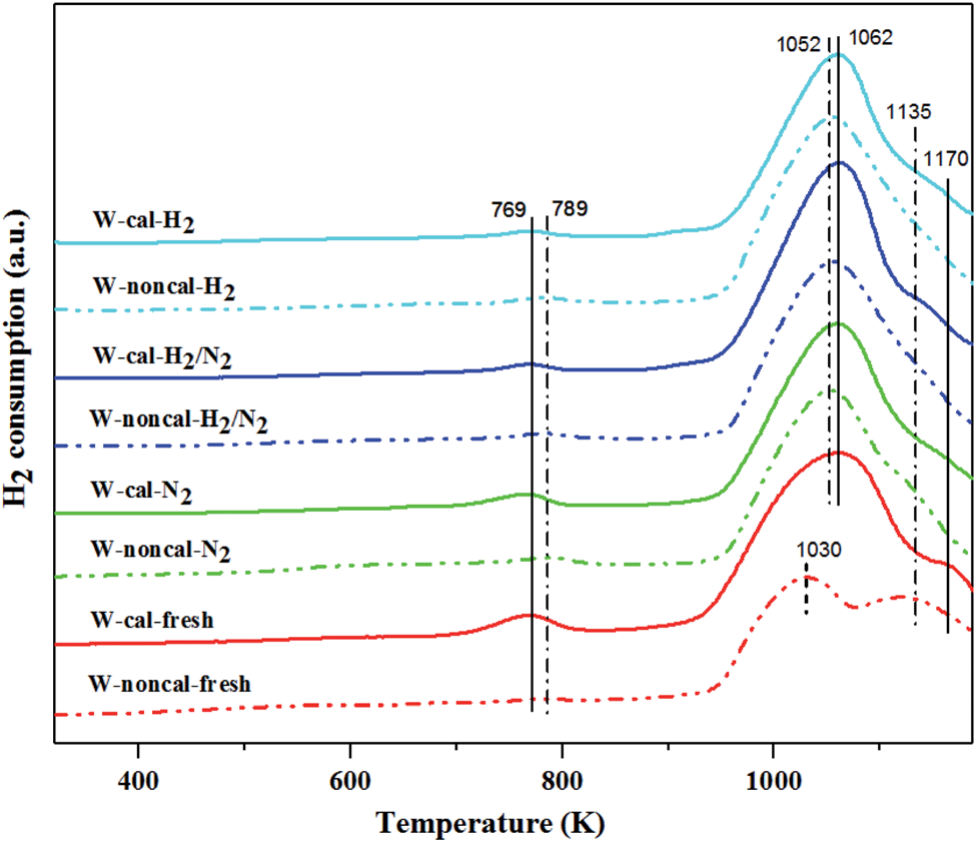
H_2_-TPR patterns of the calcined (continuous lines) and non-calcined (dotted lines) WO_3_/SiO_2_ catalysts with different gas pretreatments.

The interaction between W active species and silica support was determined by the H_2_-TPR technique. The H_2_-TPR results of the calcined and non-calcined WO_3_/SiO_2_ catalysts (Fig. 11) showed three peaks at 769–789, 1030–1062 and 1135–1170 K. The low temperature peak was attributed to the reduction of W species in octahedral coordination, and the two high temperature peaks were assigned to the reduction of the surface amorphous WO_3_ species.^10,32^ Comparing between the calcined and non-calcined WO_3_/SiO_2_ catalysts, the reduction peak at low temperature of the non-calcined catalysts appeared at higher temperature than the calcined ones, indicating that interaction of W species in octahedral coordination on silica support of the non-calcined catalysts (789 K) was stronger than the calcined catalysts (769 K). The two reduction peaks at high temperature of the non-calcined catalysts (1052 and 1135 K) appeared at lower temperature than the calcined catalysts (1062 and 1170 K), indicating that reduction of WO_3_ crystal of the non-calcined catalysts was weaker than the calcined catalysts, which reflected an improved reducibility of dispersed species.^10^ Considering the effect of gas pretreatment, the pure H_2_ pretreatment led to a boarder low temperature peak while the high temperature peaks of each type (calcined or non-calcined) catalysts were not different. Nevertheless, the H_2_ content of gas pretreatment was likely to shift the low H_2_ consumption peaks to lower temperature. Liu *et al.*^37^ mentioned that the strong acidity was partly responsible for the strong interaction between tungsten oxides and support. Therefore, these results were consistent with the NH_3_-TPD results.

**Scheme 2:**
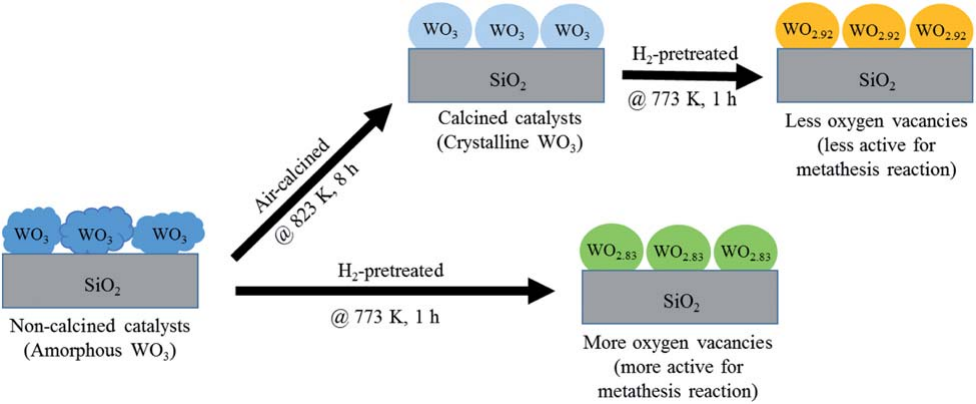
The summarized results of the non-calcined and air-calcined catalysts with H_2_ pretreatment.

According to the characterization results, it was concluded that the non-calcined catalysts had better dispersion of tungsten on the silica support (as confirmed by the BET, XRD, XPS, TEM and SEM-EDX results) than the calcined catalysts. Many researchers mentioned that higher dispersion of tungsten on the support exhibited high activity of the metathesis reaction.^14,25,35^ Additionally, the UV-Vis and Raman results also indicated the higher amount of isolated tetrahedral tungsten oxide and octahedral polytungstate species which were active species for the metathesis reaction^32^ on the non-calcined catalysts than the calcined catalysts. The NH_3_-TPD and H_2_-TPR results also showed the higher degrees of metal-support of the non-calcined catalysts due probably to the strong acidity presented on the non-calcined ones.^37^ For the comparison of gas pretreatment atmospheres, the XRD patterns of H_2_ content pretreatment atmosphere of the non-calcined catalysts revealed the formation of a WO_2.83_ phase, while those of the calcined catalysts revealed the WO_2.92_ phase. The results showed that the pure H_2_ pretreatment of the non-calcined catalyst exhibited the highest activity and performance in metathesis of ethylene and 2-butene. Therefore, it could be proposed that WO_2.83_ crystalline phase of the catalysts played an important role on the active in the metathesis reaction of ethylene and 2-butene. Nevertheless, the XPS results of pure H_2_ pretreatment of the non-calcined catalysts showed the highest amount of the W^5+^ content. Huang *et al.*^32^ proposed that W^(6−*y*)+^ (0 < *y* < 1) was the highly active centers for supported tungsten catalysts. Therefore, the improvement of the activity for metathesis reaction of ethylene and 2-butene could be adjusted by the H_2_ gas pretreatment atmosphere without the calcination with air. The concept of the summarized results is shown in Scheme 2. The catalytic performance of WO_3_/SiO_2_ catalysts could be developed without the calcination of air. The air calcination caused the stronger interaction of crystalline WO_3_ phase on the support and when reduced with H_2_ pretreatment, it exhibited the low oxygen vacancies converting crystalline WO_3_ to WO_2.92_ phase, which was less active for metathesis reaction. Whilst on the non-calcined catalysts with H_2_ pretreatment led to higher oxygen vacancies converting amorphous WO_3_ to WO_2.83_ phase. The higher oxygen vacancies could provide more active sites for metathesis reaction.

## 4. Conclusions

The effect of catalyst pretreatment atmosphere of the calcined and non-calcined WO_3_/SiO_2_ catalysts for metathesis reaction of ethylene and 2-butene to produce propylene was investigated. The gas pretreatment atmosphere consisted of pure N_2_, pure H_2_ and mixed 1 : 1 H_2_/N_2_. The catalytic activity tests were carried out at 2 pressure levels (0.1 and 2.1 MPa). At low pressure of 0.1 MPa, the gas pretreatment atmosphere for the calcined and non-calcined catalysts was not much different, while at high pressure of 2.1 MPa, the effect of pretreatment atmosphere of the non-calcined catalyst became more pronounced. The results showed that the non-calcined catalysts exhibited higher activity than the calcined catalysts. The NH_3_-TPD and H_2_-TPR results showed a stronger interaction of the catalysts and the support for the non-calcined catalysts than the calcined catalysts. Among different gas pretreatment atmospheres, pure H_2_ pretreatment of the non-calcined catalyst exhibited the highest activity and performances. The pure H_2_ pretreatment atmosphere of the non-calcined catalyst led to the phase change of tungsten species from WO_3_ to WO_2.83_, while that of the calcined catalyst converted the WO_3_ to WO_2.92_ phase, as supported by the XPS, UV-Vis and Raman results. In addition, the XPS, UV-Vis and Raman results showed that the pure H_2_ pretreatment atmosphere of the non-calcined catalyst exhibited the high isolated surface tetrahedral tungsten oxide species and W^5+^ species. The WO_2.83_ phase of the catalysts played an important role on the high activity of the WO_*x*_/SiO_2_ catalyst in metathesis reaction.

## Conflicts of interest

There are no conflicts to declare.

## Supplementary Material

## References

[cit1] Wade L. G. , Organic Chemistry, Pearson Prentice Hall, 6th edn, 2006

[cit2] Mol J. C. (2004). J. Mol. Catal. A: Chem..

[cit3] Towfighi J., Niaei A., Karimzadeh R., Saedi G. (2006). Korean J. Chem. Eng..

[cit4] Stöcker M. (1999). Microporous Mesoporous Mater..

[cit5] Cavani F., Ballarini N., Cericola A. (2007). Catal. Today.

[cit6] Li X., Zhang W., Liu S., Xu L., Han X., Bao X. (2008). J. Phys. Chem. C.

[cit7] Debecker D. P., Stoyanova M., Rodemerck U., Gaigneaux E. M. (2011). J. Mol. Catal. A: Chem..

[cit8] Spamer A., Dube T. I., Moodley D. J., van Schalkwyk C., Botha J. M. (2003). Appl. Catal., A.

[cit9] Jean-Louis Hérisson P., Chauvin Y. (1971). Die Makromolekulare Chemie.

[cit10] Zhao Q., Chen S.-L., Gao J., Xu C. (2009). Transition Met. Chem..

[cit11] Basrur A. G., Patwardhan S. R., Was S. N. (1991). J. Catal..

[cit12] Chaemchuen S., Phatanasri S., Verpoort F., Sae-ma N., Suriye K. (2012). Kinet. Catal..

[cit13] Liu H., Huang S., Zhang L., Liu S., Xin W., Xu L. (2009). Catal. Commun..

[cit14] Poovarawan N., Suriye K., Panpranot J., Limsangkass W., Santos Cadete Aires F. J., Praserthdam P. (2015). Catal. Lett..

[cit15] Wang Y., Chen Q., Yang W., Xie Z., Xu W., Huang D. (2003). Appl. Catal., A.

[cit16] Gangwal S. K., Fathi-kalajahi J., Wills G. B. (1977). Prod. R&D.

[cit17] Liu H., Tao K., Yu H., Zhou C., Ma Z., Mao D., Zhou S. (2015). C. R. Chim..

[cit18] Van Roosmalen A. J., Mol J. C. (1982). J. Catal..

[cit19] Huang S., Chen F., Liu S., Zhu Q., Zhu X., Xin W., Feng Z., Li C., Wang Q., Xu L. (2007). J. Mol. Catal. A: Chem..

[cit20] Ding K., Gulec A., Johnson A. M., Drake T. L., Wu W., Lin Y., Weitz E., Marks L. D., Stair P. C. (2016). ACS Catal..

[cit21] Lwin S., Wachs I. E. (2014). ACS Catal..

[cit22] Choung S. J., Weller S. W. (1983). Ind. Eng. Chem. Process Des. Dev..

[cit23] Westhoff R., Moulijn J. A. (1977). J. Catal..

[cit24] Zaki M. I., Fouad N. E., Mansour S. A. A., Muftah A. I. (2011). Thermochim. Acta.

[cit25] Maksasithorn S., Praserthdam P., Suriye K., Debecker D. P. (2015). Microporous Mesoporous Mater..

[cit26] Kasempremchit N., Praserthdam P., Assabumrungrat S. (2016). Korean J. Chem. Eng..

[cit27] Liu H., Zhang L., Li X., Huang S., Liu S., Xin W., Xie S., Xu L. (2009). J. Nat. Gas Chem..

[cit28] Lee J.-S., Jang I.-H., Park N.-G. (2012). J. Phys. Chem. C.

[cit29] Shpak A. P., Korduban A. M., Medvedskij M. M., Kandyba V. O. (2007). J. Electron Spectrosc. Relat. Phenom..

[cit30] Wang L., Wang Y., Cheng Y., Liu Z., Guo Q., Ha M. N., Zhao Z. (2016). J. Mater. Chem. A.

[cit31] Maksasithorn S., Debecker D. P., Praserthdam P., Panpranot J., Suriye K., Ayudhya S. K. N. (2014). Chin. J. Catal..

[cit32] Huang S., Liu S., Xin W., Bai J., Xie S., Wang Q., Xu L. (2005). J. Mol. Catal. A: Chem..

[cit33] Yang X.-L., Gao R., Dai W.-L., Fan K. (2008). J. Phys. Chem. C.

[cit34] Yue C., Zhu X., Rigutto M., Hensen E. (2015). Appl. Catal., B.

[cit35] Huang S., Liu S., Zhu Q., Zhu X., Xin W., Liu H., Feng Z., Li C., Xie S., Wang Q., Xu L. (2007). Appl. Catal., A.

[cit36] Limsangkass W., Praserthdam P., Phatanasri S., Panpranot J., Poovarawan N., Jareewatchara W., Kunjara Na Ayudhya S., Suriye K. (2014). Catal. Lett..

[cit37] Liu N., Ding S., Cui Y., Xue N., Peng L., Guo X., Ding W. (2013). Chem. Eng. Res. Des..

